# Complete Genome Sequence of Escherichia coli Siphophage Sciku

**DOI:** 10.1128/MRA.01052-19

**Published:** 2019-09-19

**Authors:** Isla Hernandez, Micah Castillo, Russell Moreland, Mei Liu, Jolene Ramsey

**Affiliations:** a Center for Phage Technology, Texas A&M University, College Station, Texas, USA; DOE Joint Genome Institute

## Abstract

Escherichia coli is a Gram-negative bacterium that is found in humans and animals as both a commensal organism and a pathogen. This report describes the isolation of Sciku, a siphophage infecting E. coli 4s, with 73 protein-coding genes. Genome comparisons suggest that Sciku is related to phages within *Guernseyvirinae*.

## ANNOUNCEMENT

Escherichia coli is a Gram-negative commensal bacterium found in the intestinal microflora of certain animals, including humans. However, not all strains are harmless, and they can cause diseases in humans, other mammals, and birds with intestinal or extraintestinal pathologies (1). Some E. coli strains carry virulence factors involved in the colonization of the intestinal tract required to develop pathology. Phage therapy is considered a viable strategy for treating E. coli infection in place of antibiotics, and to this end, we isolated bacteriophage Sciku (2).

Phage Sciku was isolated from filtered (0.2 μm) wastewater treatment sludge samples collected in College Station, Texas, using an E. coli 4s strain as the bacterial host (3). Both the phage and its host were grown aerobically at 37°C in Luria broth (BD), and standard soft-agar overlay methods were used in the isolation (4). Phage Sciku’s genomic DNA was purified with a Promega Wizard DNA cleanup system with the shotgun library preparation modifications described by Summer (5). The sequencing library was prepared with a TruSeq Nano low-throughput kit and sequenced by Illumina MiSeq with v2 500-cycle chemistry. The 565,076 total sequence reads from the index containing the phage genome were quality controlled with FastQC (www.bioinformatics.babraham.ac.uk/projects/fastqc) and assembled with SPAdes v3.5.0 at 698.9-fold contig coverage after trimming using the FastX toolkit v0.0.14 (http://hannonlab.cshl.edu/fastx_toolkit/) (6). PCR (forward primer, 5′-GGCACAGAAACCGTGTAATCT-3′; reverse primer, 5′-TGGACTCTGCCGCAAATATC-3′) and Sanger sequencing were used to close the phage genome. The Galaxy and Web Apollo instances hosted by the Center for Phage Technology (https://cpt.tamu.edu/galaxy-pub/) contain all the tools used for annotation; these were run at default parameters. For gene calling, we used GLIMMER v3.0 and MetaGeneAnnotator v1.0, along with ARAGORN v2.36 for the detection of tRNAs (7–11). Rho-independent termination sites were annotated from TransTermHP v2.09 (12). Gene function was predicted using InterProScan v5.33-72 and BLAST v2.2.31 at default settings with a maximum expectation value of 0.001 versus the NCBI nonredundant and UniProtKB Swiss-Prot and TrEMBL databases (13–15). Transmembrane domains were predicted using TMHMM v2.0 (16). Structural similarities were identified using the HHsuite v3.0 tool HHpred (multiple sequence alignment generation with HHblits using the ummiclus30_2018_08 database and modeling with the PDB_mmCIF70 database) (17). Genome-wide DNA sequence similarity was calculated with progressiveMauve v2.4.0 (18). For phage morphology, samples were negatively stained with 2% (wt/vol) uranyl acetate and viewed by transmission electron microscopy at the Texas A&M Microscopy and Imaging Center (19).

Sciku is a siphophage with a 43,130-bp genome, 50.1% G+C content, and 93.8% coding density. Our analysis assigned Sciku 73 protein-coding genes, with 34 ascribed a function, but no tRNAs. PhageTerm predicted headful packaging for Sciku, and the genome was opened in front of the small terminase subunit (20). Sciku has the highest similarity to the *Escherichia* phage VB_EcoS-Golestan (GenBank accession number MG099933) of *Guernseyvirinae*, with 56 similar unique proteins and 66.37% nucleotide identity. As seen in other *Guernseyvirinae*, Sciku has a large self-splicing intein with a Hint domain (InterProScan IPR036844) within one of its helicases (NCBI accession number QEG06907) (21).

### Data availability.

The genome sequence and associated data for phage Sciku were deposited under GenBank accession number MK931439, BioProject accession number PRJNA222858, SRA accession number SRR8893626, and BioSample accession number SAMN11414580.
